# Geochemical behavior of fluoride-rich groundwater in Markapur, Andhra Pradesh, South India

**DOI:** 10.1016/j.dib.2018.02.084

**Published:** 2018-03-08

**Authors:** Venkatayogi Sudarshan, Adimalla Narsimha, S.V.G. Das

**Affiliations:** a Department of Applied Geochemistry, University College of Science, Osmania University, Hyderabad 500007, India; b School of Environmental Science and Engineering, Chang’an University, No. 126 Yanta Road, Xi’an 710054, China; c Key Laboratory of Subsurface Hydrology and Ecological Effects in Arid Region of the Ministry of Education, Chang’an University, No. 126 Yanta Road, Xi’an 710054, Shaanxi, China; d Centre for Hydrological Education, Hyderabad 500010, India

## Abstract

Excess fluoride in drinking water has been one of the leading problem faced by the arid and semi-arid regions of the world. Significantly in India the people suffer from fluorosis comparing to other toxic elements like Arsenic etc., in drinking water. Approximately, in India the excessive fluoride in groundwater is noticed in 177 districts covering 21 states, affecting 66 million people, including 6 million children and Moreover, the latest estimation gives nearly 200 million people, from among 25 nations the world over, are affected by the deadly disease of fluorosis [1], [2], [3], [4], [5], [6], [7], [8], [9], [10], [11], [12], [13], [14]. The fluoride of the groundwater varies from 0.4 to 5.8 mg/L with a mean of 1.98 mg/L (Table 1 & 2), which indicates that the concentration of fluoride is not uniform in the study area. In general intake of small quantities of fluoride in the permissible limit of 0.5 to 1 mg/L is known to be beneficial for human health in production and maintenance of proper health. However, in India safe limit of fluoride in potable water is considered to be between 0.6 to 1.2 mg/L, less than 0.6 mg/L can cause dental caries, while higher than 1.2 mg/L leads to fluorosis [1], [2], [3], [4], [5], [6], [7], [8], [9], [10], [11], [12], [13], [14], [15], [16].

**Specifications table**

**Table:** 

Subject area	Earth Science
More specific subject area	Hydro-geochemistry
Type of data	Table, figure
How data was acquired	The fluoride concentration in water was determined electrochemically, using thermo Scientific Orion Star A214 Benchtop pH/ISE meter (9609BNWP fluoride ion-selective electrode) using the USEP ion selective electrode method [15]. This method is applicable to the measurement of fluoride in drinking water in the concentration range of 0.1–1000 mg/L. Standard fluoride solutions (0.1–10 mg/L) were prepared from a stock solution (100 mg/L) of sodium fluoride. As per experimental requirement, 2 ml of total ionic strength adjusting buffer grade III (TISAB III) was added in 20 ml of water sample. The ion meter was calibrated for a slope of −59.2±2. The composition of TISAB solution was as follows: 58 g NaCl, 4 g of CDTA (Cyclohexylene diamine tetraacetic acid) and 57 ml of glacial acetic acid per litre. Using pH/EC/TDS meter (Hanna HI 9811-5), the EC, pH and TDS of water samples were measured. Calcium, magnesium, chloride, carbonate and bicarbonate were analyzed by a titration methods. Sodium and potassium concentrations were determined using a flame photometer (Systronics, 130). Sulphate and nitrate were determined using a UV-visible spectrophotometer (Spectronic, 21, BAUSCH and LOMB).
Data format	Analyzed
Experimental factors	The bottles were soaking in 1:1 HCl for 24 h and rinsed with distilled water followed by deionized water and samples were collected after pumping out water for about 10 min to remove stagnant water from the well and then transferred and stored at 10 °C*.*
Experimental features	Determine the content levels of fluoride and other Physiochemical parameters using standard procedure.
*Data source location*	Markapur, Andhra Pradesh, South India
Data accessibility	Data is with this article

**Value of the data**

• Primarily, this data will be a guide line for the Markapur region people and scientists, hydro-geologists who works on this topic and a basic information for groundwater management studies.
• Most of the area population depends on groundwater for their daily needs, without any primary treatment and actually they do not know much about the quality of groundwater, but the nature of consequence of fluorosis is in their lifetime, it is because there no cure for it and taking safety measure of drinking water is always preferable.
• Based on this data the people who live on the current region are advised to not to drink groundwater directly for drinking purposes, if who does this constantly for a period of time will surely meet with deadly diseases of fluorosis.
• This data will be very useful to develop effective strategies for improving Markapur region water supply and provide scientific evidence for decision and management of the groundwater.

## 1 Data

**Fig. 1:**
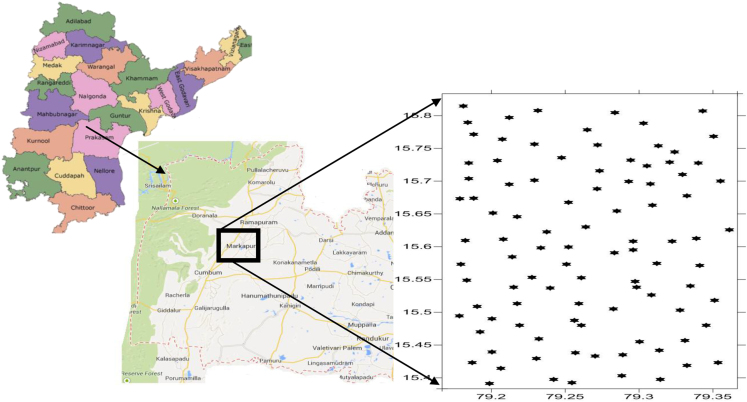
Location map of the Groundwater samples in Markapur region, Prakasam district, Andhra Pradesh, South India.

The Markapur provinces is located in central-western part of the Prakasam district (Fig. 1). The area geographically lies between the 79° 10′–79° 22′ south latitudes and 15° 35′–15° 50′ east longitudes (Fig. 1). The vast plains of Markapur and of the adjacent areas are occupied by phyllite/slate. The study area has a hot climate and classified as semi-arid with steppe type of vegetation. However, May is generally the hottest month with 45 °C with a mean minimum temperature of 27 °C, humid weather is experienced during July-November. Winds are generally light to moderate, except during the late summer and early southwest monsoon season. The average annual rainfall of the Prakasam is 798.6 mm, monthly rainfall ranges from nil in March to 182.9 mm in October. October is the wettest month of the year.

**Fig. 2:**
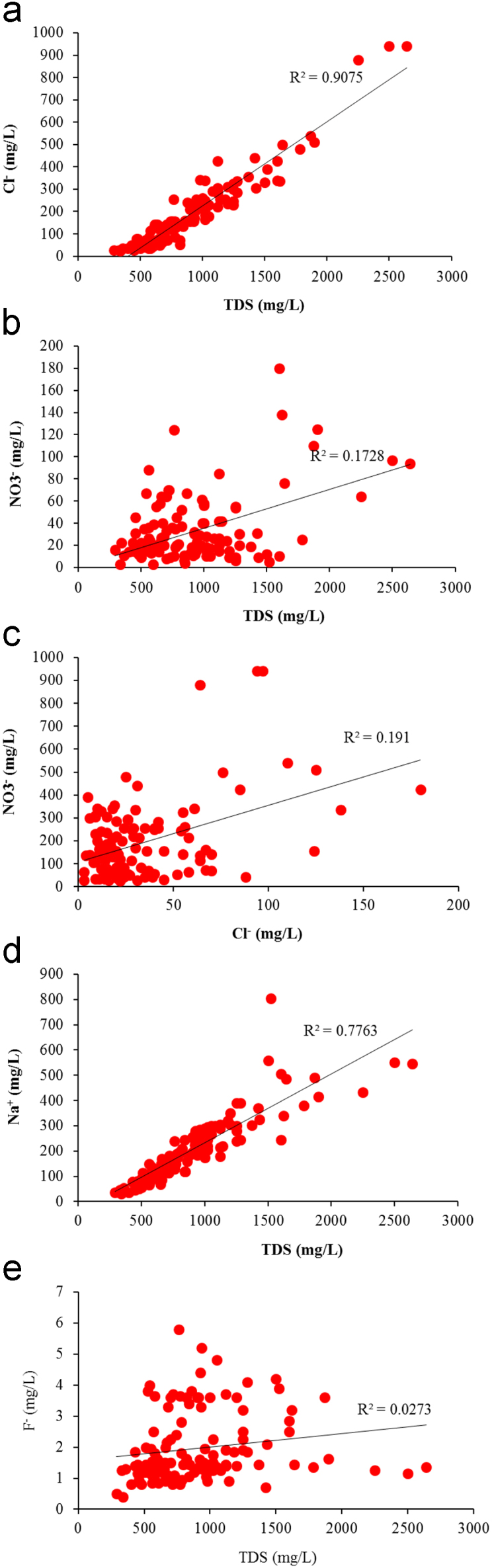
Cross plots (a) TDS vs Cl^−^, (b) TDS vs NO_3_^−^, (c) Cl^−^ vs NO_3_^−^, (d) TDS vs Na^+^, and (e) TDS vs F^−^ (TDS, Cl^−^, NO_3_^−^, Na^+^, F^−^ are expressed in mg/L).

**Fig. 3:**
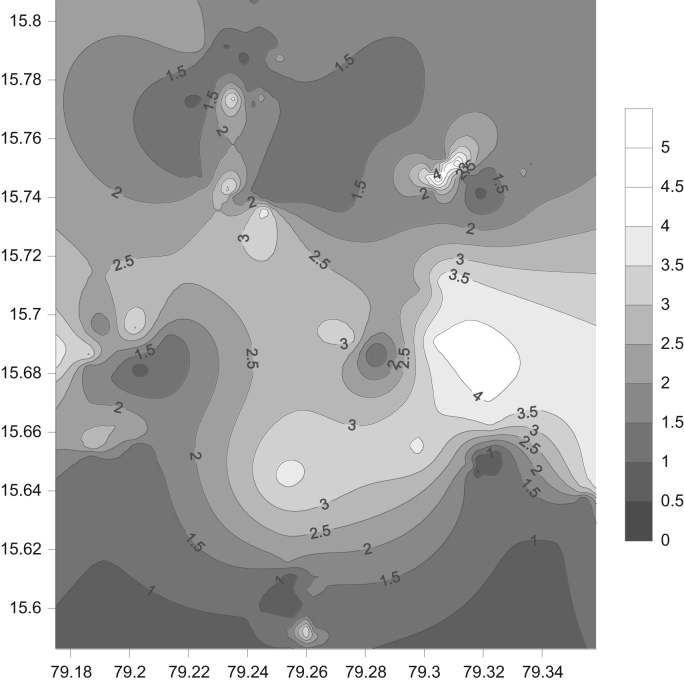
Shaded contour map of F^−^ (mg/L) for the Markapur region, Andhra Pradesh.

**Fig. 4:**
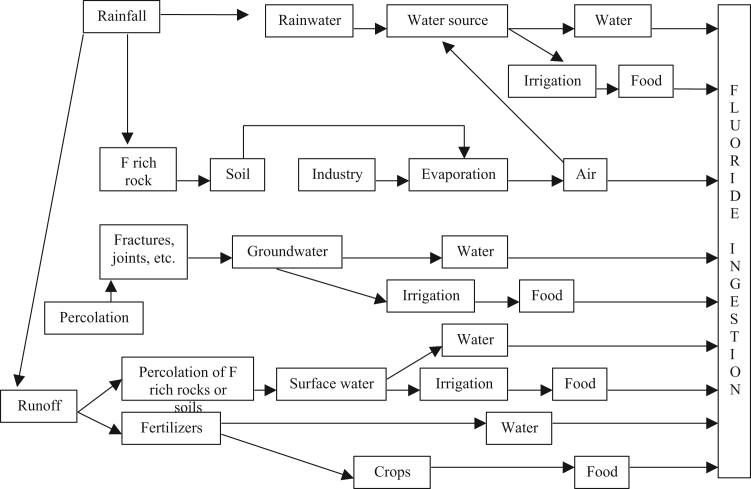
Probable transmission routes of fluoride ingestion.

Groundwater quality of Markapur, Andhra Pradesh, was studied and assessed the fluoride contaminants in groundwater. The fluoride concentrations varies from 0.4 to 5.8 mg/L and it is clear that the level of fluoride is higher in 54 groundwater locations than that of recommended upper limit by WHO (Table 1, Table 2). It is clear from the map that except for extreme north and south parts of the study area, all other areas have excess fluoride (Fig. 3). West-central part is having groundwater with highest fluoride concentration and cross plots are shown in Fig. 2. However, fluoride can gain entry into human body through different routes, probable transmission routes and its health effects are shown in Fig. 4*;* Table 3.

**Table 1:** Hydrochemical data from the Markapur, Prakasam district, Andhra Pradesh, South India.

Sample No	pH	EC	TDS	TH	Ca^2+^	Mg^2+^	Na^+^	K^+^	CO_3_^2−^	HCO_3_^−^	Cl^−^	SO_4_^2−^	F^−^	NO_3_^−^
		µS/cm	mg/L											
PDM-1	8.07	2000	1280	400	120	280	245	49	Nil	430	335	70	4.1	30
PDM-2	8.25	1800	1120	400	120	280	215	3	Nil	310	305	130	3.7	15
PDM-3	8.05	3700	2250	880	360	520	432	4	Nil	380	880	155	1.25	64
PDM-4	8.1	1520	940	340	80	260	185	2	Nil	385	200	66	1.3	23
PDM-5	8.07	3050	1870	400	80	320	490	27	Nil	545	540	120	3.6	110
PDM-6	8.11	2600	1600	620	160	460	245	88	Nil	380	425	140	2.5	180
PDM-7	8.28	1160	715	320	120	200	115	3	Nil	280	142	72	0.8	70
PDM-8	8.43	930	595	180	80	100	122	3	30	330	65	40	1.35	3
PDM-9	8.1	810	525	180	80	100	99	1	Nil	290	42	21	3.8	35
PDM-10	8.44	2000	1250	460	120	340	240	4	30	310	325	180	3.2	55
PDM-11	8.32	1410	860	320	120	200	160	16	15	365	160	47	3.8	67
PDM-12	8.71	1490	925	120	40	80	280	3	55	430	165	65	4.4	12
PDM-13	8.65	1470	910	160	25	135	260	4	55	420	165	60	3.6	11
PDM-14	8.7	1700	1050	180	80	100	300	4	60	480	180	85	4.8	17
PDM-15	8.74	1350	840	120	40	80	245	1	60	400	140	52	3.4	11
PDM-16	8.25	1950	1200	200	40	160	350	4	60	510	235	110	3.6	15
PDM-17	8.523	1230	760	80	10	70	240	1	Nil	390	120	40	5.8	10
PDM-18	8.55	1660	1020	200	40	160	285	3	40	490	185	70	1.45	15
PDM-19	8.7	1430	900	160	40	120	255	3	70	420	155	65	3.6	15
PDM-20	8.7	1810	1120	200	80	120	305	5	60	470	220	75	1.45	20
PDM-21	8.57	1500	935	120	80	40	275	3	40	440	170	35	5.2	15
PDM-22	8.7	1650	1020	160	80	80	295	4	60	330	250	115	1.75	28
PDM-23	8.73	1700	960	200	40	160	285	3	60	380	220	106	1.95	29
PDM-24	7.81	4400	2640	800	320	480	545	15	Nil	490	940	300	1.35	94
PDM-25	8.52	2300	1420	320	80	240	370	2	40	300	440	185	0.7	31
PDM-26	8.67	1620	1000	200	40	160	270	1	50	320	255	74	1.25	40
PDM-27	8.26	1800	1120	240	80	160	300	1	Nil	360	284	90	1.4	42
PDM-28	8.2	4200	2500	680	160	520	550	15	Nil	370	940	285	1.15	97
PDM-29	8.6	1740	1080	200	60	140	300	2	40	320	290	110	1.25	26
PDM-30	8.67	1250	780	200	50	150	190	2	60	330	155	62	1.8	9
PDM-31	8.75	1650	990	240	80	160	265	1	40	365	250	75	1.45	40
PDM-32	8.67	1800	1120	240	40	200	290	3	60	320	295	122	1.4	26
PDM-33	8.6	1490	920	240	80	160	225	2	55	410	170	70	1.4	30
PDM-34	8.53	1600	1000	140	40	100	290	3	40	380	215	87	3.6	8
PDM-35	8.62	1480	930	244	40	204	245	2	30	460	155	44	3.3	20
PDM-36	8.69	1300	820	160	80	80	210	3	40	460	71	51	3.6	37
PDM-37	8.6	1910	1200	280	80	200	305	3	40	400	310	109	1.4	9
PDM-38	8.4	1030	650	320	160	160	69	18	15	330	64	42	1.3	58
PDM-39	8.62	1130	720	160	80	80	182	2	30	370	71	39	3.7	70
PDM-40	8.5	610	400	220	80	140	35	4	35	230	35	13	0.8	14
PDM-41	8.55	1220	770	200	40	160	184	3	25	380	130	35	3.65	18
PDM-42	8.56	870	560	260	80	180	74	2	25	265	60	42	1.55	36
PDM-43	8.43	1300	820	200	80	120	193	1	20	520	53	20	1.63	52
PDM-44	8.53	850	550	240	80	160	80	0.5	40	310	57	16	1.95	15
PDM-45	8.23	830	510	240	120	120	76	2	Nil	340	42	12	1.45	16
PDM-46	7.95	1230	770	280	140	140	150	1	Nil	215	255	36	0.8	5
PDM-47	7.18	1590	1000	360	120	240	175	32	Nil	360	260	55	1.45	6
PDM-48	8.15	700	450	240	120	120	46	2	Nil	275	32	8	1.15	5
PDM-49	8.3	860	560	220	40	180	90	7	10	350	43	10	1.25	22
PDM-50	8.5	900	580	140	40	100	136	6	30	370	43	6	3.65	13
PDM-51	8.32	890	580	260	60	200	120	13	5	365	35	9	1.45	21
PDM-52	8.52	870	560	240	80	160	83	7	30	350	43	6	1.1	24
PDM-53	8.28	1060	680	240	60	180	124	14	Nil	410	64	13	3.3	20
PDM-54	8.23	1020	650	270	40	230	102	11	Nil	360	78	20	1.4	22
PDM-55	8.25	870	565	240	60	180	76	19	Nil	360	36	6	1.5	22
Sample No	pH	EC	TDS	TH	Ca^2+^	Mg^2+^	Na^+^	K^+^	CO_3_^2−^	HCO_3_^−^	Cl^−^	SO_4_^2−^	F^−^	NO_3_^−^
PDM-56	8.51	860	560	270	40	230	67	9	30	320	57	10	0.85	26
PDM-57	8.42	870	560	240	60	180	77	20	30	340	50	9	1.1	18
PDM-58	8.41	840	520	220	60	160	77	12	20	310	43	8	1.2	19
PDM-59	8.32	890	580	200	40	160	108	5	15	330	57	14	1.8	16
PDM-60	8.32	760	500	170	40	130	92	6	10	290	50	10	1.25	22
PDM-61	8.57	760	470	200	40	160	80	1	40	260	78	10	0.85	15
PDM-62	8.54	520	330	160	60	100	47	1	30	190	28	17	1.25	3
PDM-63	8.26	550	340	200	60	140	30	1	Nil	210	25	12	0.4	22
PDM-64	8.33	890	290	140	80	60	35	3	15	160	28	17	0.5	16
PDM-65	8.19	560	360	160	120	40	53	1	Nil	210	35	13	1.3	11
PDM-66	8.39	840	510	160	40	120	115		25	300	60	18	2	20
PDM-67	8.5	660	430	160	60	100	78	1	40	210	50	15	1.4	22
PDM-68	8.05	930	600	180	40	140	129	1	Nil	395	43	4	1.63	39
PDM-69	8.04	1340	845	240	40	200	120	3	Nil	460	135	12	1.45	4
PDM-70	8.46	900	580	220	40	180	90	24	20	260	115	13	1.1	14
PDM-71	8.07	1020	660	260	80	180	85	3	Nil	310	120	13	1.05	22
PDM-72	8.32	740	480	240	40	200	50	8	10	220	78	9	0.8	12
PDM-73	8.23	2900	1780	500	160	340	380	54	Nil	380	480	230	1.35	25
PDM-74	8.7	3100	1900	440	120	320	415	11	50	460	510	190	1.63	125
PDM-75	8.23	2700	1620	440	160	280	340	4	Nil	420	335	235	3.2	138
PDM-76	8.15	2050	1250	360	160	200	300	2	Nil	450	245	200	2.5	54
PDM-77	8.3	1650	1020	280	120	160	245	3	10	420	165	155	2.25	18
PDM-78	8.34	2350	1430	360	120	240	325	4	20	420	305	185	2.1	9
PDM-79	8.44	710	460	180	80	100	75	3	30	250	50	26	0.95	20
PDM-80	8.35	2050	1250	320	120	200	280	4	20	280	300	215	1.9	6
PDM-81	8.37	2250	1370	360	120	240	302	13	20	260	355	210	1.45	19
PDM-82	8.04	2650	1640	240	80	160	485	5	Nil	440	500	145	1.45	76
PDM-83	8.5	2040	1280	160	40	120	390	3	40	525	285	81	1.85	20
PDM-84	8.63	2020	1250	160	60	100	390	3	40	600	230	78	2.25	9
PDM-85	8.35	1880	1180	240	40	200	320	4	15	500	255	65	1.85	24
PDM-86	8.21	1680	1050	240	60	180	267	7	Nil	440	230	65	1.35	10
PDM-87	8.32	2630	1600	200	40	160	505	2	10	680	340	130	2.85	10
PDM-88	8.2	2430	1500	200	40	160	557	3	Nil	600	330	130	4.2	12
PDM-89	8.48	1530	950	260	80	180	225	9	20	320	255	68	1.5	29
PDM-90	8.46	1280	795	200	80	120	195	6	30	380	142	34	1.45	21
PDM-91	8.03	1550	970	420	80	340	237	10	Nil	390	220	62	1.05	20
PDM-92	8.31	1160	730	200	60	140	166	12	10	360	130	32	1.1	9
PDM-93	7.8	1340	840	200	60	140	118	12	Nil	415	140	47	1.05	6
PDM-94	8.6	2450	1520	120	40	80	805	2	40	530	390	118	3.9	5
PDM-95	9.32	1020	655	160	80	80	150	3	10	280	130	29	1.5	20
PDM-96	8.24	660	430	160	80	80	75	3	Nil	240	43	14	1.85	15
PDM-97	8.34	1000	640	200	80	120	130	3	15	260	142	21	1.2	14
PDM-98	8.15	1450	900	280	80	200	195	5	Nil	260	210	80	1.3	18
PDM-99	8.07	1080	700	240	80	160	132	5	Nil	350	114	17	2.25	64
PDM-100	8.06	1630	1020	360	160	200	203	5	Nil	180	340	94	1.5	18
PDM-101	8.51	830	540	200	80	120	95	3	30	300	72	10	4	67
PDM-102	8.44	1110	700	200	80	120	155	6	20	350	106	25	1.5	8
PDM-103	8	1810	1120	480	200	280	178	5	Nil	130	425	95	1.9	85
PDM-104	8.28	1060	660	240	80	160	127	7	Nil	280	135	32	0.9	64
PDM-105	8.25	1060	680	240	120	120	124	4	Nil	330	115	21	1.1	28
PDM-106	8.15	950	610	200	80	120	120	4	Nil	245	130	25	0.85	19
PDM-107	8.46	1260	785	240	80	160	173	7	30	380	128	40	0.95	20
PDM-108	8.27	1180	745	200	80	120	175	4	Nil	280	155	52	2.4	36
PDM-109	7.75	1840	1140	400	240	160	220	27	Nil	360	255	94	0.9	42
PDM-110	8.55	1230	760	320	160	160	130	6	40	360	156	26	0.9	124
PDM-111	7.86	1620	1020	330	130	200	220	7	Nil	410	200	85	1.25	10
PDM-112	8.15	1580	980	320	120	200	205	5	Nil	190	341	92	0.9	61
Sample No	pH	EC	TDS	TH	Ca^2+^	Mg^2+^	Na^+^	K^+^	CO_3_^2−^	HCO_3_^−^	Cl^−^	SO_4_^2−^	F^−^	NO_3_^−^
PDM-113	8.32	880	570	200	80	120	106	5	5	280	85	23	2.5	24
PDM-114	8.19	1430	880	280	120	160	184	15	Nil	210	240	82	1.2	12
PDM-115	8.47	920	560	120	80	40	150	1	30	340	43	20	1.45	88
PDM-116	8.56	1020	660	120	60	60	170		40	385	50	18	2	27
PDM-117	8.54	1050	670	160	40	120	163	1	40	390	57	20	2.15	40
PDM-118	8	1090	700	240	100	140	134	3	Nil	380	85	25	3.6	33
PDM-119	8.41	720	450	220	80	140	58	2	20	300	28	4	1.45	31
PDM-120	8.22	780	500	260	100	160	55	2	Nil	320	35	5	1.2	9
PDM-121	8.59	1250	780	200	40	160	184	13	40	450	85	25	2.8	20
PDM-122	8.3	1470	920	200	40	160	242	6	10	250	215	130	1.6	32
PDM-123	8.21	960	620	265	120	145	92	5	Nil	115	141	144	1.45	55

PDM-Indicates: Prakasam District Markapur Region.

**Table 2:** Groundwater samples of the Markapur, Prakasam district, Andhra Pradesh, South India exceeding the permissible limits prescribed by WHO (2004) and ISI (1993) for drinking purpose.

Water quality parameters	Indian Standard (ISI 10500, 1993)		WHO International Standards (2004)		Range in the Markapur region
	Highest desirable	Max. permissible	Most desirable limit	Max. allowable limit	
pH	6.5–8.5	6.5–9.5	6.5	8.5	7.18–9.32
EC	–	–	1400	–	520–4400
TDS	500	2000	500	1500	290–2640
TH	300	600	100	500	80–880
CO_3_^2−^	–	–	–	–	5–70
HCO_3_^−^	–	–	–	–	115–680
Cl^−^	250	1000	200	600	25–940
NO_3_^−^	–	45	–	45	3–180
F^−^	0.6	1.2	0.5	1.5	0.4–5.8
Ca^2+^	75	200	75	200	10–360
Mg^2+^	30	100	50	150	40–520
K^+^	–	–	–	12	0.5–88
Na^+^	–	200	–	200	30–805

**Table 3:** Effects of fluoride ingestion on human health (Adimalla and Venkatayogi, 2017).

Fluoride concentration (mg/L)	Effect on human health
<0.5	Conducive to dental caries
0.5 to 1.5	Promotes development of strong bones and teeth
1.5 to 4.0	Promotes dental fluorosis in children
>4.0	Promotes dental and skeletal fluorosis
>10	Crippling skeletal fluorosis, possibly cancer

## 2 Experimental design, materials, and methods

123 groundwater samples have been collected from bore wells/tube wells in the study area. The bottles were soaking in 1:1 HCl for 24 h and rinsed with distilled water followed by deionized water and samples were collected after pumping out water for about 10 min to remove stagnant water from the well and then transferred and stored at 10 °C. All collected groundwater samples were separately labeled with sample ID starting from PDM-1 to PDM-123, and were transferred to the laboratory and analyzed in the laboratory for analysis of major anionic and cationic constituents using standard methods APHA (1995). The pH, electrical conductivity (EC), total dissolved solids (TDS), were analyzed on the site using pH/EC/TDS meter (Hanna HI 9811-5). Total hardness (TH) was measured by titration method using standard hydrochloric acid and standard EDTA solution. Calcium (Ca^2+^) and magnesium (Mg^*+*^) were determined titrimetrically using standard EDTA. Sodium (Na^*+*^) and potassium (K^*+*^) concentrations were determined using Flame photometer (Systronics, 130). Chloride (Cl^*−*^*) was determined by standard AgNO*_*3*_ titration. Bicarbonate (HCO_3_^−^) and carbonate (CO_3_^2−^) by titration with HCl. Sulphate (SO_4_^2−^) and Nitrate (NO_3_^−^) were determined by using UV-visible spectrophotometer (Spectronic, 21, BAUSCH and LOMB). The fluoride concentration in water was determined electrochemically, using thermo Scientific Orion Star A214 Benchtop pH/ISE meter (9609BNWP fluoride ion-selective electrode) using the USEP ion selective electrode method. This method is applicable to the measurement of fluoride in drinking water in the concentration range of 0.1–1000 mg/L. Standard fluoride solutions (0.1–10 mg/L) were prepared from a stock solution (100 mg/L) of sodium fluoride. As per experimental requirement, 2 ml of total ionic strength adjusting buffer grade III (TISAB III) was added in 20 ml of water sample. The ion meter was calibrated for a slope of −59.2 ± 2. The composition of TISAB solution was as follows: 58 g NaCl, 4 g of CDTA (Cyclohexylene diamine tetraacetic acid) and 57 ml of glacial acetic acid per litre. Eventually, the accuracy of all chemical analyses was verified by calculating ion-charge balance between cations (Ca^2+^, Mg^2+^, Na^+^ and K^+^) and anions (HCO_3_^−^, Cl^−^,SO_4_^2−^, NO_3_^−^ and F^−^) as (cations - anions)/(cations+anions)X100, all 123 groundwater samples were less than the accepted limit of ±10% and samples.
